# Poly[bis(μ-purin-9-ido-κ^2^ *N* ^7^:*N* ^9^)zinc]

**DOI:** 10.1107/S1600536812011245

**Published:** 2012-03-21

**Authors:** A. Cadiau, K. Adil

**Affiliations:** a LUNAM Université, Université du Maine, CNRS UMR 6283, Institut des Molécules et des Matériaux du Mans, Avenue Olivier Messiaen, 72085 Le Mans CEDEX 9, France

## Abstract

In the title compound, [Zn(C_5_H_3_N_4_)_2_], the Zn^II^ cation is in a nearly regular tetra­hedral coordination by purinate ligands. Each purinate ligand chelates two Zn^II^ cations through two imidazole N atoms of the purinate anion ligand, leading to the formation of a three-dimensional network.

## Related literature

For common applications of hybrid materials, see: Cui *et al.* (2012 ▶); Horcajada *et al.* (2012 ▶); Li *et al.* (2012 ▶); Stock & Biswas (2012 ▶); Suh *et al.* (2012 ▶); Sumida *et al.* (2012 ▶); Yoon *et al.* (2012 ▶). For characteristic zinc–nitro­gen distances in metal-organic framework compounds, see: Cadiau *et al.* (2011 ▶).
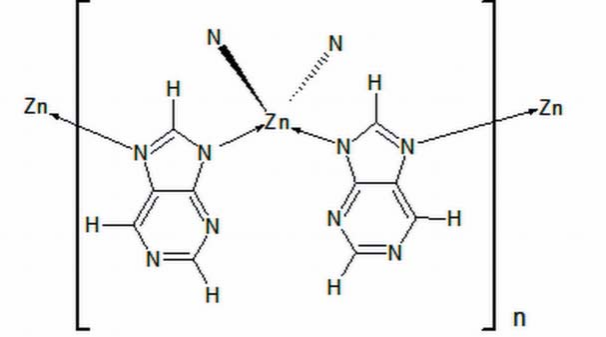


## Experimental

### 

#### Crystal data

[Zn(C_5_H_3_N_4_)_2_]
*M* *_r_* = 303.60
Orthorhombic, 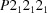

*a* = 9.2332 (5) Å
*b* = 10.1337 (6) Å
*c* = 12.4186 (6) Å
*V* = 1161.96 (11) Å^3^
*Z* = 4
Mo *K*α radiation
μ = 2.11 mm^−1^
*T* = 296 K
0.45 × 0.31 × 0.07 mm

#### Data collection

Bruker APEXII Quazar CCD diffractometer
Absorption correction: multi-scan (*SADABS*; Sheldrick, 1996 ▶) *T*_min_ = 0.580, *T*_max_ = 0.746
4522 measured reflections
2336 independent reflections
2129 reflections with *I* > 2σ(*I*)
*R*_int_ = 0.026

#### Refinement

*R*[*F* ^2^ > 2σ(*F* ^2^)] = 0.024
*wR*(*F* ^2^) = 0.053
*S* = 1.01
2336 reflections
172 parameters
H-atom parameters constrained
Δρ_max_ = 0.42 e Å^−3^
Δρ_min_ = −0.26 e Å^−3^
Absolute structure: Flack (1983 ▶)
Flack parameter: 0.030 (13)

### 

Data collection: *APEX2* (Bruker, 2007 ▶); cell refinement: *SAINT-Plus* (Bruker, 2007 ▶); data reduction: *SAINT-Plus*; program(s) used to solve structure: *SHELXTL* (Sheldrick, 2008 ▶); program(s) used to refine structure: *SHELXTL*; molecular graphics: *DIAMOND* (Brandenburg, 2009 ▶); software used to prepare material for publication: *SHELXTL*.

## Supplementary Material

Crystal structure: contains datablock(s) global. DOI: 10.1107/S1600536812011245/vn2034sup1.cif

Structure factors: contains datablock(s) I. DOI: 10.1107/S1600536812011245/vn2034Isup2.hkl

Additional supplementary materials:  crystallographic information; 3D view; checkCIF report

## Figures and Tables

**Table 1:** Selected bond lengths (Å)

Zn1—N1	2.010 (2)
Zn1—N2	2.006 (2)
Zn1—N3	1.994 (2)
Zn1—N5	1.983 (2)

**Fig. 1.**
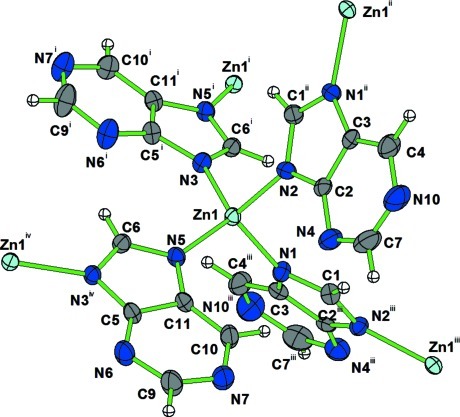
A view of the structure of the title compound showing the coordination environment of the Zn atom; displacement ellipsoids are drawn at the 50% probability level. Symmetry codes: (i) –x, –0.5+y, 1.5–z; (ii) –0.5+x, 1.5-y, 1–z; (iii) 0.5+x, 1.5–y, 1–z; (iv) –x, 0.5+y, 1.5–z

**Fig. 2.**
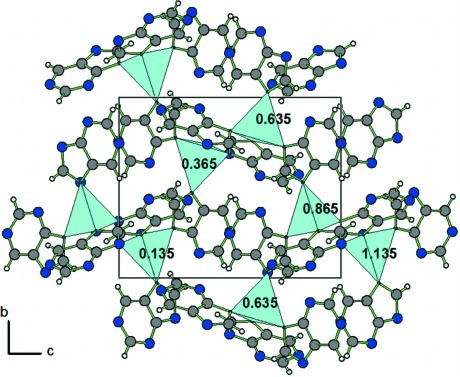
Projection of the structure along the a axis showing the three-dimensional network. The numbers refer to the x-coordinates of the ZnII cations.

## supplementary crystallographic information

### Comment

The emerging class of hybrids materials known as Metal-Organic Frameworks (MOFs) has attracted much attention because of their enormous variety of interesting structural topologies (Stock & Biswas, 2012) and wide potential applications as functional materials, such as gas storage (Suh *et al.*, 2012; Sumida *et al.*, 2012), separation (Li *et al.*, 2012), catalysis (Yoon *et al.*, 2012) and luminescence (Cui *et al.*, 2012). Moreover, there is a growing interest in MOFs for biological application (Horcajada *et al.*, 2012) such as the drug controlled release or using MOFs based on endogenous linkers (nucleobases and amino acids). We report here on the synthesis and crystal structure of a new three-dimensional zinc MOFs material elaborated from purinate linkers.

The asymmetric unit of the title compound consists of one Zn^II^ cation and two non-equivalent purine molecules. Fig. 1 displays in a symmetry-expanded view the full coordination sphere of the Zn atom. Selected geometric parameters are given in Table 1. Zn^II^ are linked to four N atoms from two purinate anions to form quite regular tetrahedra. The coordination Zn—N bond lengths range from 1.983 (2) to 2.009 (3) Å which are in a good agreement with the literature (Cadiau *et al.*, 2011). The structure of Zn(C_5_H_3_N_4_)_2_ compound can be described as originating from deprotonated purinate anions (C_5_N_4_H_3_^-^) linked to Zn^II^ cations in order to generate a three-dimensional network as is shown in Fig.2.

### Experimental

Chemicals have been purchased from commercial sources and were used as received without further purification. The title compound was prepared under hydrothermal conditions at 393 K for 48 h using Teflon-lined autoclaves from a started mixture of zinc fluoride (Alfa Aesar), purine (Sigma-Aldrich) and deionized water under the following conditions: ZnF_2_ (0.067 g, 0.65 mmol), C_5_H_4_N_4_ (0.480 g, 4 mmol), H_2_O (5 mL). The resulting crystalline product was washed with water and dried in air. Needle yellow crystals suitable for single-crystal X-ray diffraction were selected using an optical microscope.

### Refinement

Hydrogen atoms bonded to the ligands were positioned geometrically and refined using a riding model with C—H = 0.93 Å. These hydrogen atoms were assigned isotropic thermal parameters and *U*_iso_(H) = 1.2×*U*_eq_(C).

### Crystal data

**Table:**

[Zn(C_5_H_3_N_4_)_2_]	*F*(000) = 608
*M**_r_* = 303.60	*D*_x_ = 1.735 Mg m^−^^3^
Orthorhombic, *P*2_1_2_1_2_1_	Mo *K*α radiation, λ = 0.71073 Å
Hall symbol: P 2ac 2ab	Cell parameters from 896 reflections
*a* = 9.2332 (5) Å	θ = 2–11°
*b* = 10.1337 (6) Å	µ = 2.11 mm^−^^1^
*c* = 12.4186 (6) Å	*T* = 296 K
*V* = 1161.96 (11) Å^3^	Needle, yellow
*Z* = 4	0.45 × 0.31 × 0.07 mm

### Data collection

**Table:**

Bruker APEXII Quazar CCD diffractometer	2336 independent reflections
Radiation source: ImuS microsource	2129 reflections with *I* > 2σ(*I*)
Mirror monochromator	*R*_int_ = 0.026
ω scans	θ_max_ = 29.0°, θ_min_ = 3.3°
Absorption correction: multi-scan (*SADABS*; Sheldrick, 1996)	*h* = −12→11
*T*_min_ = 0.580, *T*_max_ = 0.746	*k* = −10→13
4522 measured reflections	*l* = −15→11

### Refinement

**Table:**

Refinement on *F*^2^	Secondary atom site location: difference Fourier map
Least-squares matrix: full	Hydrogen site location: inferred from neighbouring sites
*R*[*F*^2^ > 2σ(*F*^2^)] = 0.024	H-atom parameters constrained
*wR*(*F*^2^) = 0.053	*w* = 1/[σ^2^(*F*_o_^2^) + (0.014*P*)^2^] where *P* = (*F*_o_^2^ + 2*F*_c_^2^)/3
*S* = 1.01	(Δ/σ)_max_ = 0.001
2336 reflections	Δρ_max_ = 0.42 e Å^−^^3^
172 parameters	Δρ_min_ = −0.26 e Å^−^^3^
0 restraints	Absolute structure: Flack (1983)
Primary atom site location: structure-invariant direct methods	Flack parameter: 0.030 (13)

### Special details

**Table:**

Geometry. All e.s.d.'s (except the e.s.d. in the dihedral angle between two l.s. planes) are estimated using the full covariance matrix. The cell e.s.d.'s are taken into account individually in the estimation of e.s.d.'s in distances, angles and torsion angles; correlations between e.s.d.'s in cell parameters are only used when they are defined by crystal symmetry. An approximate (isotropic) treatment of cell e.s.d.'s is used for estimating e.s.d.'s involving l.s. planes.
Refinement. Refinement of *F*^2^ against ALL reflections. The weighted *R*-factor *wR* and goodness of fit *S* are based on *F*^2^, conventional *R*-factors *R* are based on *F*, with *F* set to zero for negative *F*^2^. The threshold expression of *F*^2^ > σ(*F*^2^) is used only for calculating *R*-factors(gt) *etc*. and is not relevant to the choice of reflections for refinement. *R*-factors based on *F*^2^ are statistically about twice as large as those based on *F*, and *R*- factors based on ALL data will be even larger.

### Fractional atomic coordinates and isotropic or equivalent isotropic displacement parameters (Å^2^)

**Table:**

	*x*	*y*	*z*	*U*_iso_*/*U*_eq_	
Zn1	0.05827 (3)	0.83956 (3)	0.63470 (2)	0.01682 (8)	
N1	0.2523 (2)	0.7592 (2)	0.60162 (16)	0.0211 (5)	
N2	−0.0615 (2)	0.81227 (19)	0.50179 (15)	0.0202 (5)	
N3	−0.0333 (2)	0.7247 (2)	0.74592 (16)	0.0200 (5)	
N4	0.0678 (3)	0.9343 (2)	0.3640 (2)	0.0310 (5)	
N5	0.0775 (2)	1.0294 (2)	0.67090 (16)	0.0198 (5)	
N6	0.1801 (3)	1.3634 (2)	0.63818 (19)	0.0335 (6)	
N7	0.3055 (3)	1.2441 (3)	0.4986 (2)	0.0356 (6)	
C1	0.3147 (3)	0.7551 (3)	0.5048 (2)	0.0216 (6)	
H1	0.2743	0.7967	0.4452	0.026*	
C2	−0.0407 (3)	0.8578 (2)	0.39896 (19)	0.0189 (5)	
C3	0.3449 (3)	0.6859 (2)	0.66539 (19)	0.0214 (6)	
C4	−0.1555 (3)	0.8505 (4)	0.2285 (2)	0.0337 (7)	
H4	−0.2284	0.8209	0.1827	0.040*	
C5	0.1262 (3)	1.2481 (3)	0.6690 (2)	0.0214 (6)	
C6	0.0102 (3)	1.0927 (3)	0.7501 (2)	0.0201 (6)	
H6	−0.0487	1.0494	0.7995	0.024*	
C7	0.0542 (4)	0.9665 (3)	0.2603 (3)	0.0399 (8)	
H7	0.1256	1.0216	0.2324	0.048*	
N10	−0.0503 (4)	0.9289 (3)	0.19110 (19)	0.0436 (7)	
C9	0.2668 (4)	1.3533 (3)	0.5524 (2)	0.0386 (8)	
H9	0.3052	1.4321	0.5267	0.046*	
C10	0.2476 (3)	1.1293 (3)	0.5314 (2)	0.0275 (7)	
H10	0.2709	1.0512	0.4961	0.033*	
C11	0.1542 (3)	1.1274 (2)	0.61731 (19)	0.0197 (6)	

### Atomic displacement parameters (Å^2^)

**Table:**

	*U*^11^	*U*^22^	*U*^33^	*U*^12^	*U*^13^	*U*^23^
Zn1	0.01916 (15)	0.01367 (13)	0.01764 (14)	0.00023 (14)	0.00213 (13)	−0.00095 (13)
N1	0.0207 (12)	0.0231 (12)	0.0196 (12)	0.0036 (10)	0.0009 (10)	−0.0011 (10)
N2	0.0180 (11)	0.0203 (12)	0.0222 (11)	−0.0017 (11)	−0.0017 (10)	−0.0009 (9)
N3	0.0238 (14)	0.0147 (11)	0.0215 (11)	0.0011 (10)	0.0041 (11)	0.0020 (9)
N4	0.0270 (13)	0.0319 (13)	0.0342 (13)	−0.0027 (11)	0.0072 (15)	0.0007 (12)
N5	0.0242 (13)	0.0150 (11)	0.0203 (10)	−0.0006 (10)	0.0010 (10)	−0.0004 (9)
N6	0.0483 (15)	0.0243 (13)	0.0281 (12)	−0.0110 (11)	0.0090 (13)	−0.0048 (12)
N7	0.0438 (16)	0.0312 (14)	0.0316 (12)	−0.0104 (12)	0.0115 (12)	−0.0034 (11)
C1	0.0251 (15)	0.0198 (13)	0.0200 (12)	0.0025 (11)	−0.0016 (12)	0.0008 (11)
C2	0.0159 (14)	0.0167 (13)	0.0241 (12)	0.0052 (11)	0.0026 (11)	−0.0017 (11)
C3	0.0190 (14)	0.0233 (15)	0.0220 (14)	−0.0035 (12)	−0.0041 (12)	−0.0031 (11)
C4	0.0288 (17)	0.052 (2)	0.0204 (14)	0.0018 (17)	0.0006 (13)	0.0007 (16)
C5	0.0294 (16)	0.0172 (14)	0.0175 (13)	−0.0034 (12)	−0.0012 (12)	−0.0016 (11)
C6	0.0212 (14)	0.0187 (13)	0.0204 (13)	−0.0001 (11)	0.0027 (11)	0.0000 (10)
C7	0.0324 (18)	0.0397 (18)	0.0477 (19)	−0.0041 (18)	0.015 (2)	0.0052 (15)
N10	0.0399 (17)	0.0639 (19)	0.0270 (14)	−0.0063 (17)	0.0099 (15)	0.0064 (13)
C9	0.056 (2)	0.0285 (18)	0.0313 (16)	−0.0165 (17)	0.0122 (16)	−0.0052 (14)
C10	0.0344 (18)	0.0237 (17)	0.0244 (15)	−0.0009 (12)	0.0039 (14)	−0.0061 (12)
C11	0.0245 (15)	0.0180 (14)	0.0167 (13)	−0.0019 (10)	−0.0027 (12)	−0.0004 (10)

### Geometric parameters (Å, º)

**Table:**

Zn1—N1	2.010 (2)	C1—N2^iii^	1.333 (4)
Zn1—N2	2.006 (2)	C1—H1	0.9300
Zn1—N3	1.994 (2)	C2—C3^i^	1.397 (4)
Zn1—N5	1.983 (2)	C3—C4^iii^	1.368 (3)
N1—C1	1.335 (4)	C3—C2^iii^	1.397 (4)
N1—C3	1.382 (3)	C4—N10	1.338 (4)
N2—C1^i^	1.333 (4)	C4—C3^i^	1.368 (3)
N2—C2	1.371 (3)	C4—H4	0.9300
N3—C6^ii^	1.356 (3)	C5—N3^iv^	1.381 (3)
N3—C5^ii^	1.381 (3)	C5—C11	1.405 (3)
N4—C7	1.335 (4)	C6—N3^iv^	1.356 (3)
N4—C2	1.339 (3)	C6—H6	0.9300
N5—C6	1.328 (3)	C7—N10	1.348 (4)
N5—C11	1.390 (3)	C7—H7	0.9300
N6—C5	1.327 (3)	C9—H9	0.9300
N6—C9	1.337 (4)	C10—C11	1.372 (4)
N7—C9	1.341 (4)	C10—H10	0.9300
N7—C10	1.343 (3)		
			
N5—Zn1—N3	116.54 (9)	N2—C2—C3^i^	108.7 (2)
N5—Zn1—N2	111.68 (8)	C4^iii^—C3—N1	134.0 (3)
N3—Zn1—N2	104.81 (8)	C4^iii^—C3—C2^iii^	117.9 (2)
N5—Zn1—N1	111.08 (9)	N1—C3—C2^iii^	108.1 (2)
N3—Zn1—N1	106.44 (9)	N10—C4—C3^i^	119.5 (3)
N2—Zn1—N1	105.50 (9)	N10—C4—H4	120.3
C1—N1—C3	103.4 (2)	C3^i^—C4—H4	120.3
C1—N1—Zn1	125.61 (19)	N6—C5—N3^iv^	127.2 (2)
C3—N1—Zn1	130.69 (17)	N6—C5—C11	124.4 (2)
C1^i^—N2—C2	103.6 (2)	N3^iv^—C5—C11	108.3 (2)
C1^i^—N2—Zn1	126.39 (18)	N5—C6—N3^iv^	115.5 (3)
C2—N2—Zn1	129.99 (18)	N5—C6—H6	122.3
C6^ii^—N3—C5^ii^	103.8 (2)	N3^iv^—C6—H6	122.3
C6^ii^—N3—Zn1	122.31 (19)	N4—C7—N10	127.8 (3)
C5^ii^—N3—Zn1	133.86 (17)	N4—C7—H7	116.1
C7—N4—C2	112.6 (3)	N10—C7—H7	116.1
C6—N5—C11	104.3 (2)	C4—N10—C7	117.8 (2)
C6—N5—Zn1	126.50 (18)	N6—C9—N7	128.3 (3)
C11—N5—Zn1	129.08 (17)	N6—C9—H9	115.9
C5—N6—C9	112.8 (2)	N7—C9—H9	115.9
C9—N7—C10	117.2 (3)	N7—C10—C11	119.9 (2)
N2^iii^—C1—N1	116.2 (3)	N7—C10—H10	120.1
N2^iii^—C1—H1	121.9	C11—C10—H10	120.1
N1—C1—H1	121.9	C10—C11—N5	134.6 (2)
N4—C2—N2	127.0 (2)	C10—C11—C5	117.4 (2)
N4—C2—C3^i^	124.4 (2)	N5—C11—C5	108.0 (2)

Symmetry codes: (i) *x*−1/2, −*y*+3/2, −*z*+1; (ii) −*x*, *y*−1/2, −*z*+3/2; (iii) *x*+1/2, −*y*+3/2, −*z*+1; (iv) −*x*, *y*+1/2, −*z*+3/2.
